# New Insights Into Gut-Bacteria-Derived Indole and Its Derivatives in Intestinal and Liver Diseases

**DOI:** 10.3389/fphar.2021.769501

**Published:** 2021-12-13

**Authors:** Xiaojing Li, Binbin Zhang, Yiyang Hu, Yu Zhao

**Affiliations:** ^1^ Key Laboratory of Liver and Kidney Diseases (Ministry of Education), Institute of Liver Diseases, Shanghai Key Laboratory of Traditional Chinese Clinical Medicine, Shuguang Hospital Affiliated to Shanghai University of Traditional Chinese Medicine, Shanghai, China; ^2^ Institute of Clinical Pharmacology, Shuguang Hospital Affiliated to Shanghai University of Traditional Chinese Medicine, Shanghai, China

**Keywords:** indole, indole derivates, intestinal inflammation, liver diseases, tryptophan metabolites

## Abstract

The interaction between host and microorganism widely affects the immune and metabolic status. Indole and its derivatives are metabolites produced by the metabolism of tryptophan catalyzed by intestinal microorganisms. By activating nuclear receptors, regulating intestinal hormones, and affecting the biological effects of bacteria as signaling molecules, indole and its derivatives maintain intestinal homeostasis and impact liver metabolism and the immune response, which shows good therapeutic prospects. We reviewed recent studies on indole and its derivatives, including related metabolism, the influence of diets and intestinal commensal bacteria, and the targets and mechanisms in pathological conditions, especially progress in therapeutic strategies. New research insights into indoles will facilitate a better understanding of their druggability and application in intestinal and liver diseases.

## Introduction

The crosstalk between the host and the gut microbiota has been widely studied in recent decades. Individuals carry more than 1,000 distinct bacterial species; each bacteria has significant genomic differences, which leads to variable protein expression and metabolite formation (Belizario and Faintuch, 2018). The gut microbiota and microbiota-derived small molecules, such as indole and its derivatives, interact with the host and exert a variety of local and heterotopic biological effects by circulating in the plasma (Schroeder and Backhed, 2016).

The liver and intestine are closely related through the liver–gut axis, and the disorder of intestinal–liver interaction plays an important role in the pathogenesis of gastrointestinal and liver diseases (Tripathi et al., 2018). Intestinal microbiota dysbiosis and impaired intestinal barrier function lead to the translocation of microbiota and its products, which triggers the innate and adaptive immune system, induces chronic inflammation, and leads to liver damage and systemic metabolic disorders (McLaughlin et al., 2017; Chen and Tian, 2020). Gut microbiota is involved in the bioconversion of indoles from tryptophan (Trp), which is an essential amino acid derived entirely from the diet. In the gastrointestinal tract, indole compounds can accumulate to millimolar concentrations (Liu et al., 2020); changes in food intake and gut microbiota also have a great impact on the metabolic concentration of indole in the intestine.

Recently, as a part of microbiotherapy, indole and its derivates were extensively explored in terms of antifungal, anti-platelet, and antioxidant effects in various diseases (Mucha et al., 2021). Accumulating shreds of evidence have demonstrated that indole and its derivatives derived from the intestine exert a profound effect on the intestinal barrier function and intestinal immunity (Alexeev et al., 2018; Scott et al., 2020). Indole and its derivatives can enter the liver through the circulation for further catalysis and affect liver metabolism and immune response as important intercellular signal molecules (Banoglu et al., 2001; Hwang et al., 2013; Brunius et al., 2016). In this review, we gathered the current knowledge of the gut microbiota–derived indoles, with a focus on related gut metabolic pathways and enzymes, the influence of dietary intake, the pathophysiological changes, and the prospect of potential biotechnological applications in intestinal and liver diseases (Table 1).

**TABLE 1:** Catalyze enzymes, functions, and potential side effects of microbial-origin tryptophan metabolites.

Metabolites	Enzymes (gene)	Model	Function	References	Potential side effect
Indole	Tryptophanase (TnaA)	DSS-induced colitis mice	Increases tight junction and adheres junction-associated molecule expression in colonic epithelial cells	Shimada et al. (2013)	Enhances blood pressure and colon permeability Huc et al. (2018)
		*In vitro* culture of *Escherichia coli*	Intestinal bacteria have a biphasic chemotaxis effect on indole	Yang et al. (2020a)	Enhances IL-22 production; may promote tumor progression Hernandez et al. (2018)
		Eimeria-induced chicken intestinal inflammatory	Keeping the balance between Treg cell and Th17 cells *via* AHR activities	Kim et al. (2019)	Promotes *Clostridioides difficile* proliferation Darkoh et al. (2019)
		Human enterocyte cell line HCT-8	Increases epithelial-cell tight-junction protein expression *via* AHR activation; decreases TNF-α–mediated NF-κB activation, IL-8 expression and increases IL-10 expression	Bansal et al. (2010)	Prolonged exposure to indole inhibits GLP-1 secretion Chimerel et al. (2014)
		GLUTag cells	Regulates gut motility and stimulates GLP-1 secretion	Chimerel et al. (2014)	Activated AHR induces insulin resistance and promotes NAFLD susceptibility Kerley-Hamilton et al. (2012); Kumar et al. (2021)
		HFD-fed mice	Increases the expression of tight junction proteins and intestinal mucosa	Jennis et al. (2018)	100–250 μM indole exhibits an AhR antagonist activity
		LPS-treated precision-cut liver slices	Reduces pro-inflammatory mediators	Beaumont et al. (2018)	—
		LPS-injected mice	Downregulates liver pro-inflammatory gene expression through an NLRP3-dependent pathway in Kupffer cells		—
IAA: indole-3-acetate	Tryptophan monooxygenase and indole-3-acetamide hydrolase	HepG2 and AML12 cells	Attenuates lipid loading–induced inflammatory responses; reduces Fasn and SREBP-1c expression *via* AhR activation	Krishnan et al. (2018)	—
		Macrophages	Reduces pro-inflammatory cytokine production in free fatty acid and LPS-treated RAW 264.7 macrophages; decreases MCP-1–treated bone marrow–derived macrophage migration		
		HFD-induced NAFLD mice	Attenuates hepatic lipogenesis and oxidative and inflammatory stress	Ji et al. (2019)	
IAld: indole-3-aldehyde	—	*Lactobacillus reuteri D8* treated intestinal organoid and lamina propria lymphocyte co-cultured system and DSS-induced colitis mice	Stimulates lamina propria lymphocytes to secrete IL-22 partially dependent on AhR; induces STAT3 phosphorylation to accelerate the intestinal epithelial proliferation	Hou et al. (2018)	—
		DSS-induced colitis mice	Inhibits myosin IIA and erzin activation; maintaining the integrity of the intestinal barrier in an AhR-dependent way	Scott et al. (2020)	
IPyA: indole-3-pyruvate	Aromatic amino acid aminotransferases (ArATs)	T cell transfer colitis mice model	Serving as microbiota-derived murine AHR agonists; alters the composition of mesenteric lymph node dendritic cells and decreasing lamina propria Th1 cells of the colon differentiation; attenuates Th1 cytokine production and increases IL-10 production	Aoki et al. (2018)	Decreases the production of the proinflammatory cytokine IL-1β and promotes the parasite *Trypanosoma brucei* to evade the host immune response McGettrick et al. (2016)
ILA: indole-3-lactate	fldH phenyllactate dehydrogenase (fldH)	Germ-free mice or mice lacking DP IELs	Reprogram intraepithelial CD4^+^ T cells into immunoregulatory T cells *via* AHR activities	Cervantes-Barragan et al. (2017)	Disrupts epithelial autophagy, increases colon injury susceptibility, and promotes colitis progression Fan et al. (2020)
IA: indole-3-acrylate	Phenyllacetate dehydratase (fldAIBC)	Colonic spheroids	Increasing Muc 2 expression and promoting goblet cell function; inducing AHR target gene CYP1A1 expression	Wlodarska et al. (2017)	
		LPS-treated BMDM and colonic spheroid co-culture system	Enhancing IL-10 expression and reducing TNF-α		
		LPS-stimulated human peripheral blood mononuclear cells	Inhibiting IL 6 and IL-1β secretion; promoting antioxidant and anti-inflammatory immune responses partly *via* NRF2-ARE pathway activation		
IPA: indole-3-propionate	acyl-CoA dehydrogenase (AcdA)	IFN-γ–treated T84 intestinal cell monolayers	Reducing human intestinal epithelial cell permeability and inflammation *via* decreasing the expression of GLUT5	Jennis et al. (2018)	IPA served as a PXR agonist, but PXR activation can induce CD36 expression to promote steatosis in human hepatic cells Zhou et al. (2008); Bitter et al. (2015)
		Nr1i2^−/−^ mice and Nr1i2^+/+^ mice with or without indomethacin treatment	Regulating mucosal integrity through upregulating junctional protein expression and downregulating TNF-α *via* PXR activation	Venkatesh et al. (2014)	Aggravating CCL4-induced liver fibrosis Liu et al. (2021)
		HFD-fed mice	Reducing gut permeability	Jennis et al. (2018)	—
Indican: indoxyl-3-sulfate	Liver CYP2E1 and sulfotransferases	Th17 differentiation model	Serving as potent endogenous agonist for AHR	Hwang et al. (2013)	Serving as an extensively studied uremic solute Leong and Sirich (2016)
		HFD-fed mice	Decreased indoxyl sulfate–repressed miR-181a and miR-181b expression in adipocytes and contributed to the progression of obesity, IR, and WAT inflammation	Virtue et al. (2019)	
indigo	—	HFD-induced insulin resistance and NAFLD mice	Improving intestinal barrier permeability and reducing endotoxemia *via* increasing IL-10 and IL-22 production; ameliorating immune-mediated inflammatory changes in the intestine and liver	Lin et al. (2019)	—
Tryptamine	Trp decarboxylase enzyme (TrpD gene)	IFN-γ–treated T84 intestinal cell monolayers	Reducing human intestinal epithelial cell permeability *via* AhR activation	Jennis et al. (2018)	Lower concentration (50 μM) of tryptamine promote 2,3,7,8-tetrachlordibenzo-p-dioxin mediated AhR activation Jin et al. (2014)
		MCP-1–treated BMDMs	Decreasing macrophage migration	Krishnan et al. (2018)	
		Germ-free mice colonized with engineered *Bacteroides thetaiotaomicron*	Goblet cell activation and mucus release *via* 5-HT4R activation	Bhattarai et al. (2020)	

AHR, aryl hydrocarbon receptor; PXR, pregnane X receptor; 5-HT4R, 5-HT, 4 receptor; BMDMs, bone-marrow-derived macrophages; STAT3, signal transducer and activator transcription 3; LPS, lipopolysaccharide; NAFLD, nonalcoholic fatty liver disease; TLR-4, Toll like receptor 4; TNF, tumor necrosis factor; IR, insulin resistance; WAT, white adipose tissue; Fasn, fatty acid synthase; SREBP-1c, sterol regulatory element-binding protein-1c; GLUT5, fructose transporter SLC2A5; NRF2-ARE, NF-E2-related factor 2-antioxidant response element.

## Metabolism of Indole and its Derivates: Pathways and Enzymes

### Intestinal Trp Metabolism

**FIGURE 1:**
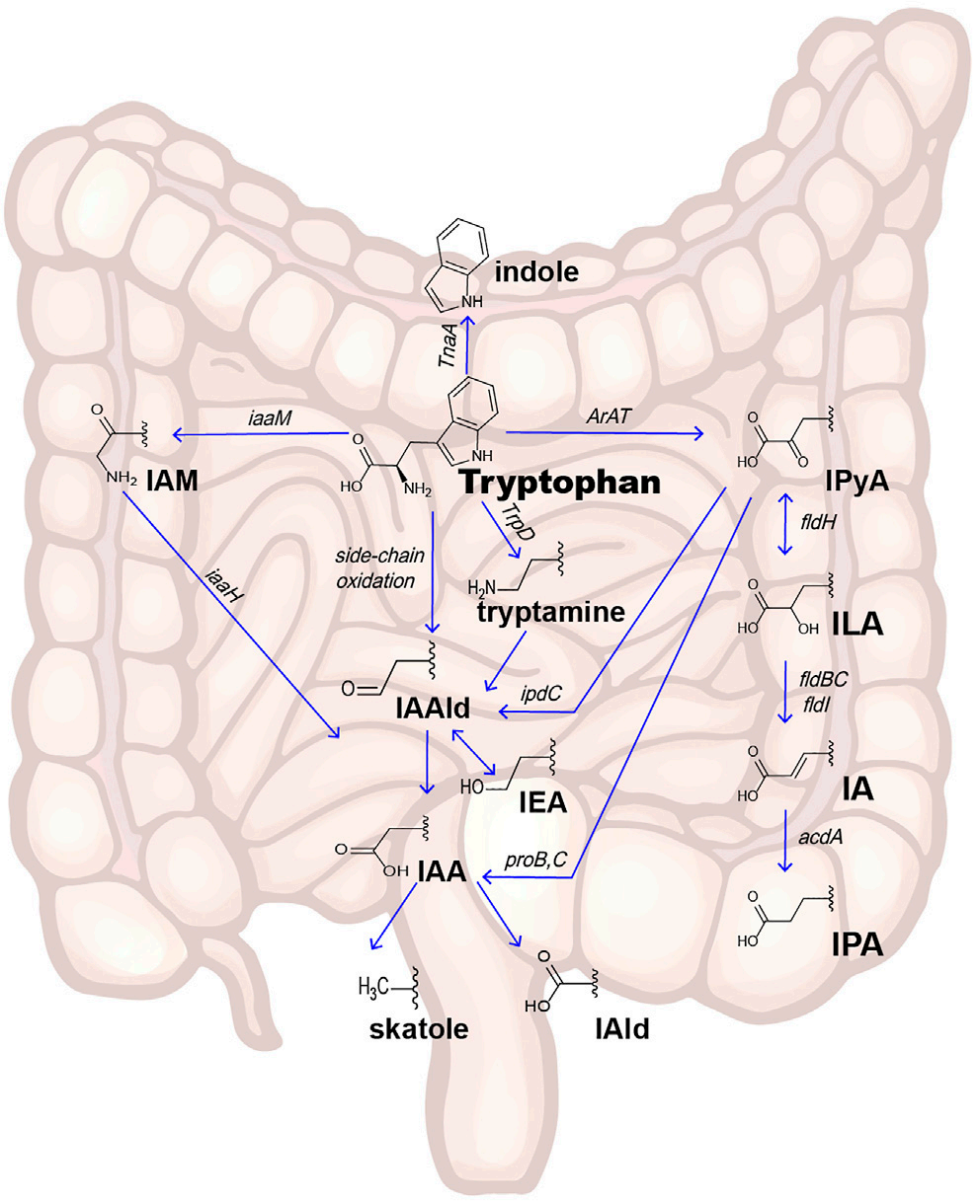
Pathways of intestinal Trp metabolism. Indole and its derivatives are derived from the metabolism of Trp by gut microorganisms. There are three main pathways in intestinal microorganism–derivated Trp metabolism: the Trp-Indole pathway, Trp-IPyA-ILA-IA-IPA pathway, and Trp-IAA-Skatole or IAld pathway. Abbreviations: IPyA, indole-3-pyruvate; ILA, indole-3-lactate; IA, indole-3-acrylate; IPA, indole-3-propionate; IAAld, indole-3-acetaldehyde; IAA, indole-3-acetate; IAld, indole-3-aldehyde; IAM, indole-3-acetamine; IEA, indole-3-ethanol; *TnaA* gene, encode Tryptophanase; *iaaM* gene, encode Tryptophan 2-monooxygenase; *iaaH* gene, encode indole-3-acetamide hydrolase; *TrpD* gene: encode Tryptophan decarboxylase enzyme; *ArAT* gene, encode aromatic amino acid aminotransferase; *fldH* gene, encode phenyllactate dehydrogenase; *fldBC* gene, encode (R)-phenyllactyl-CoA dehydratase alfa and beta subunits; *acdA* gene, encode acyl-CoA dehydrogenase; *ipdC* gene, encode Indole-3-pyruvate decarboxylase.

Indole and its derivatives are derived from the metabolism of Trp by gut microorganisms. Trp is an essential aromatic amino acid that cannot be synthesized endogenously; therefore, the exogenous dietary source of Trp intake is decisive. A small portion of the ingested Trp serves as a substrate to synthesize proteins, and the remainder is metabolized by endogenous host cells (the kynurenine pathway and serotonin pathway) (Gostner et al., 2020) or intestinal microorganisms (indole and its derivatives pathway) (Figure 1 **).**

#### Trp→Indole Pathway

As the most abundant microbial Trp catabolites, more than 85 gram-positive and gram-negative species are known to hydrolyze Trp to indole by tryptophanase (Lee and Lee, 2010). *Escherichia coli* is one of the most widely studied intestinal indole-producing bacteria. The expression of tryptophanase in *E. coli* is controlled by the Trp operon, which consists of a promoter, a short peptide regulatory gene, and two structural genes, *TnaA* and *TnaB* (Yanofsky et al., 1991). Trp is imported into cells by *TnaB* and then reversibly catalyzed by *TnaA* to indole, pyruvate, and ammonia (Watanabe and Snell, 1972; Ma et al., 2018; Yang W. et al., 2020). Because eukaryotes cannot encode *TnaA*, this reaction occurs only in bacteria (Lee and Lee, 2010). The fecal concentration of indole was reported to be in a wide range (from 0.30 to 6.64 millimole in healthy adults), which indicates that there are very large individual differences in indole metabolism (Darkoh et al., 2015). The concentration of indole depends on the amount of exogenous Trp; under Trp-rich conditions, *TnaA* can be synthesized to convert more Trp into indole by *E. coli* (Li and Young, 2013). Intriguingly, though some bacteria, such as *Aeromonas salmonicida*, *Pseudovibrio* sp., etc., have a *TnaA* gene homolog, they cannot synthesize indole (Lee and Lee, 2010).

#### Trp→Tryptamine Pathway

Tryptamine is a tryptophan-derived monoamine, and two known commensal microorganisms, *Ruminococcus gnavus* and *Clostridium sporogenes*, convert Trp into tryptamine by the action of Trp decarboxylase enzyme (TrpD). In healthy individuals, the presence of TrpD homologs is at least 10% according to the analysis of the samples from the NIH Human Microbiome Project (HMP), which indicates that the production of tryptamine by gut microbiota could be prevalent in humans (Williams et al., 2014). Transplanting human gut microbiota to germ-free mice increases tryptamine concentrations by nearly 200-fold (Marcobal et al., 2013).

#### Trp→Indole-3-Pyruvate→Indole-3-Lactate→Indole-3-Acrylate→Indole-3-Propionate Pathway

IPyA is converted from Trp by the presence of the aromatic amino acid aminotransferase (ArAT). IPyA is a precursor of ILA, and phenyllactate dehydrogenase (*fldH*) is involved in this reduction reaction. Through dehydrating, bacterial species containing phenyllactate dehydratase (*fldBC*) along with its activator *fldI* convert ILA to IA. IA can be further converted into IPA by acyl-CoA dehydrogenase (*AcdA*), which is the final product of reductive Trp metabolism (Dodd et al., 2017).

A series of studies have reported that the intestinal microbiota plays a key role in this process. ArAT is an enzyme that is phylogenetically conserved in many bacterial species, including *Lactobacilli* and *Clostridium sporogenes* (Zelante et al., 2013; Aoki et al., 2018). Fifty-one species of *Bifidobacterium* are reported to convert Trp into ILA (Van Benschoten, 1979). Another group of probiotic *Lactobacillus* spp. convert Trp to ILA by an indolelactic acid dehydrogenase (ILDH) (Wilck et al., 2017; Lee et al., 2018). Several *Peptostreptococcus* spp. and *Clostridium* spp. promote the synthesis of IA and IPA due to the homologous gene cluster of phenyllactate dehydratase *fldAIBC* (Dodd et al., 2017; Wlodarska et al., 2017); *fldC*, especially, is essential for the reductive metabolism of Trp by gut bacteria, and its mutant can block the dehydration of ILA and increase the level of the other upstream metabolites. Bacteria such as *Peptostreptococcus stomatis*, which lacks the initiator *fldI* but encodes the phenyllactate dehydratase *fldABC* gene cluster, produce a lower level of IA (Wlodarska et al., 2017). Other bacteria, such as *Lechevalieria aerocolonigenes*, can synthesize IPA through Trp deamination, which is catalyzed by amino acid oxidase (Nishizawa et al., 2005).

#### Trp→Indole-3-Acetate →Methylindole (Skatole) or Indole-3-Aldehyde Pathway

IAA is one of the Trp degraded molecules by fungi and bacteria. In human fecal samples, the mean concentration of IAA is 5 nM/g (Lamas et al., 2016). IAA biosynthetic includes multiple coexisting pathways; at least five major Trp-dependent metabolic precursors to IAA have been postulated and identified (Zelante et al., 2021), namely IPyA, indole-3-acetamine (IAM), indole-3-acetonitrile (IAN), tryptamine, and the tryptophan side-chain oxidation pathway (only demonstrated in *Pseudomonas fluorescens*). Tryptamine and IPyA are the most prevalent synthetic precursors in bacteria (Zhang et al., 2019; Miyagi et al., 2020). Tryptophan monooxygenase (TMO, encoded by *IaaM* gene) catalyzes Trp to IAM, and then the indole-3-acetamide hydrolase (encoded by *IaaH* gene) covers IAM to IAA (Tsavkelova et al., 2012). In the IPyA-IAA pathway, IPyA decarboxylase is the key enzyme (encoded by *ipdC* gene) (Malhotra and Srivastava, 2008). IPyA is catalyzed by this enzyme to IAAld, and then IAAld can be converted into IAA *via* decarboxylation. In addition, candidate genes in the genome include pyruvate: ferredoxin oxidoreductases B and C (*ProB* and *ProC*) and putative flavin-containing monooxygenases (FMOs) are also involved in converting IPyA to IAA (Dai et al., 2013; Dodd et al., 2017). In addition to bacteria-derived IAA synthetic pathways, it was recently reported that interleukin-4-induced gene 1, which encodes a protein with l-amino acid activity that has a bias for catalyzing L-aromatic amino acids, could catalyze Trp to IAA in host cells (Zhang et al., 2020).

Skatole and IAld are the terminal products of Trp-IAA degradation. The concentration of precursor IAA could be the rate-limiting factor of skatole synthesis (Yokoyama and Carlson, 1979). Less than 0.01% of the total intestinal microbiota, mainly the *Clostridium* and *Bacteroides* genera, catalyzes the steps from IAA to skatole (Wesoly and Weiler, 2012). The fecal skatole concentration in healthy humans is 5 μg/g feces, whereas its level can increase to 80–100 μg/g feces in digestive disorder patients (Yokoyama and Carlson, 1979). IAA can also be oxidized to IAld through peroxidase-catalyzed aerobic oxidations, even under strong oxidation conditions; this step occurs spontaneously *in vitro* (Zhang et al., 2020). IAld is produced in a reduced number of species that belong to the Firmicutes phyla, such as *Lactobacillus acidophilus*, *Lactobacillus murinus*, and *Lactobacillus reuteri.* IAld is produced *via* several pathways, of which the IPyA route is the main pathway for IAld synthesis from Trp (Trp→IPyA→IAld) (Zelante et al., 2013; Zelante et al., 2021).

### Metabolism of Indoles in Host

The absorption of indole and its derivatives through the intestinal epithelium is due to the ability to freely diffuse through lipid membranes (Pinero-Fernandez et al., 2011). A variety of metabolic enzymes, such as cytochrome P450 (CYP450), exist in human intestinal epithelial cells to facilitate the absorption of indoles (Gillam et al., 2000). Then, the indole compounds afflux into the liver through the portal vein and undergo further metabolism.

**FIGURE 2:**
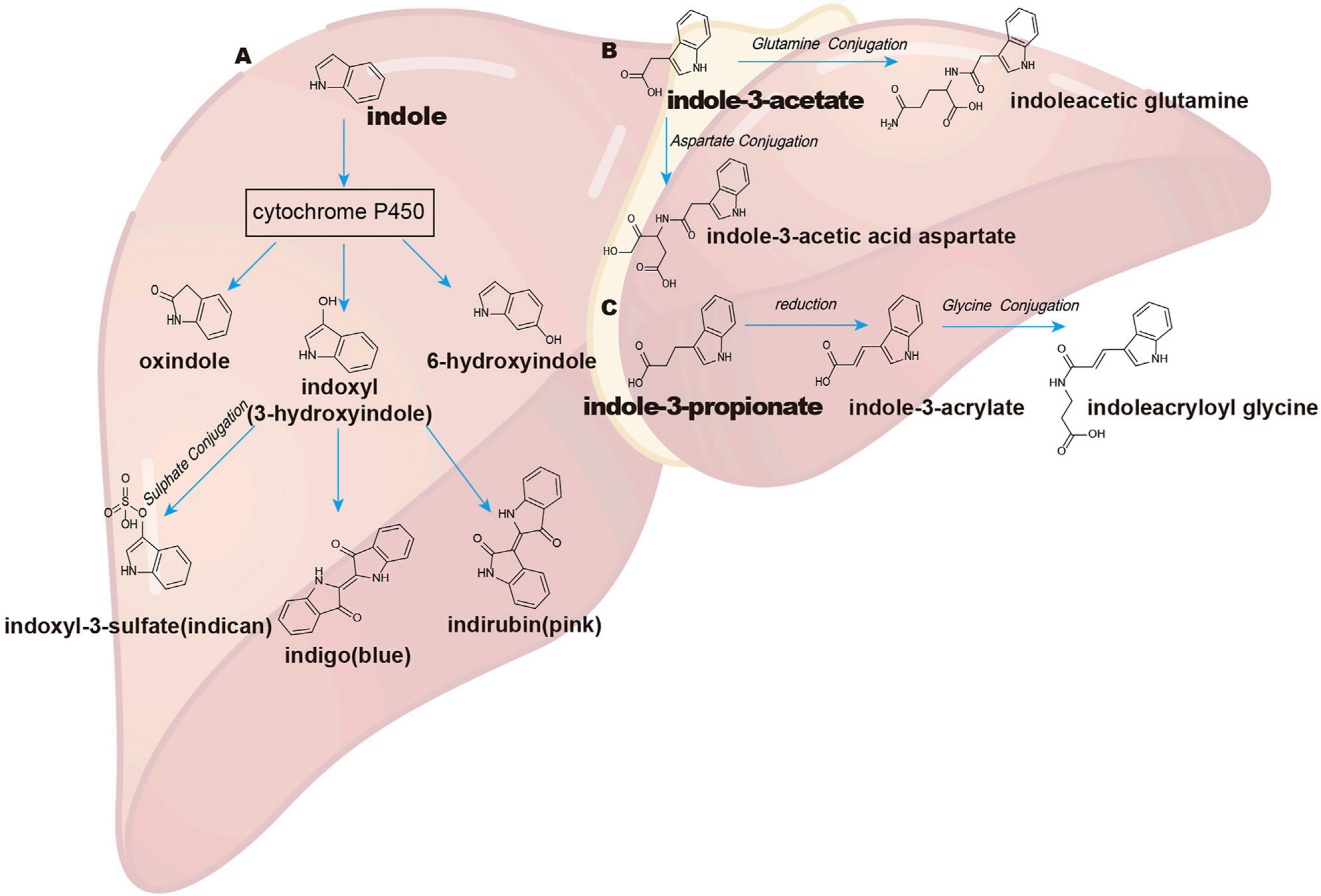
Pathways of indole and indole derivate metabolism in the liver. **(A)** indole; **(B)** indole-3-acetate (IAA); **(C)** indole-3-propionate (IPA). The absorption of indole and its derivatives through the intestinal epithelium and their further metabolism by liver CYP450 and sulfotransferase enzymes and conjugation with some other amino acids like glutamine and glycine and, finally, excretion by the kidney.

Indole metabolism in the liver has been widely studied. Initially, indole is absorbed and oxidized by microsomal CYP450 isozymes (especially the CYP2E1 isoform) to indoxyl and indican (Banoglu et al., 2001). CYP2A6 was also reported to oxidize indole at the C-2, C-3, and C-6 positions to synthesize oxindole, indoxyl (3-hydroxyindole), and 6-hydroxyindole, respectively (Gillam et al., 2000). Hepatic phase II drug metabolism of indoxyl is conjugated by sulfotransferases to generate indoxyl-3-sulfate (I3S), which is finally excreted by the kidney (Banoglu et al., 2001; Banoglu and King, 2002). As a uremic toxin, indoxyl sulfate is extensively studied in uremia (Lano et al., 2020; Wang et al., 2020) (Figure 2A).

Apart from indole, liver CYP450, especially CYP1A, CYP2A, and CYP2E1, plays a key role in skatole metabolism and produces seven known skatole metabolites (indole-3-carbinol, 3-hydroxy-3-methyloxindole, 3-methyloxyindole, 3-hydroxy-3-methylindolenine, 5-hydroxy-3-methylindole, 6-hydroxy-3-methylindole, and 2-aminoacetophenone) (Brunius et al., 2016). In the liver, IAA can be oxidized by peroxidase to cytotoxic species (Folkes et al., 1999) or be further combined with glutamine to synthesize indoleacetic glutamine (Keszthelyi et al., 2009; Gao et al., 2018) (Figure 2B). IPA can be converted into IA and further conjugated with glycine to produce indoleacryloyl glycine in the liver (Smith et al., 1968; Gao et al., 2018; Ulaszewska et al., 2020) (Figure 2C). Tryptamine can be deaminated by monoamine oxidases A and B, which are highly expressed in the colonic epithelium and liver (Jones, 1982; Bhattarai et al., 2018).

## Influence of Dietary Intake and Ingredients

Dietary intake has a wide influence on gut microbiota and the related indole metabolism. Microbial Trp metabolism activity can be reduced when alternative energy substrates are available. Indole is much lower in animals fed a high-nonstarch polysaccharide diet (Knarreborg et al., 2016). This finding is consistent with the report in 1919 that glucose repressed indole biosynthesis (Wyeth, 1919). Recently, some researchers have found that IPA was the metabolite most significantly and consistently related to the intake of fiber and inversely associated with the risk of type 2 diabetes (de Mello et al., 2017). The associated mechanism can be partially explained by the host functional LCT variant and fiber intake interaction on the gut bacteria shifting tryptophan metabolism (de Mello et al., 2017; Qi et al., 2021). In addition to carbohydrates, dietary cholesterol was reported to drive the formation of nonalcoholic fatty liver disease–associated hepatocellular carcinoma by inducing changes in mouse gut microbiota and metabolites. Dietary intake of high-cholesterol formula increases serum taurocholic acid but reduces IPA concentration in mice and can be prevented by cholesterol suppression therapy (Zhang et al., 2021). Compared with a low-fat diet, the high-fat diet–fed mice exhibited a significant depletion of IA and tryptamine in the liver and cecum (Krishnan et al., 2018). High salt intake also has an effect on consuming *Lactobacillus murine* and reducing fecal levels of ILA and IAA (Wilck et al., 2017). Excess alcoholic consumption exhibits an increasing level of IAM in the ceca of mice (Liu et al., 2019). It is worth noting that some dietary intervention studies did not specify the ratio of indole content in dietary formulas. However, indole, as an interspecies and interkingdom signaling molecule, is also widely present in plants. Therefore, some dietary intervention studies cannot exclude the effect of dietary intake factors on the benefit of increasing the concentration of indole and its derivatives.

## The Effects of Indole and Its Derivatives

### Activation of AhR

**FIGURE 3:**
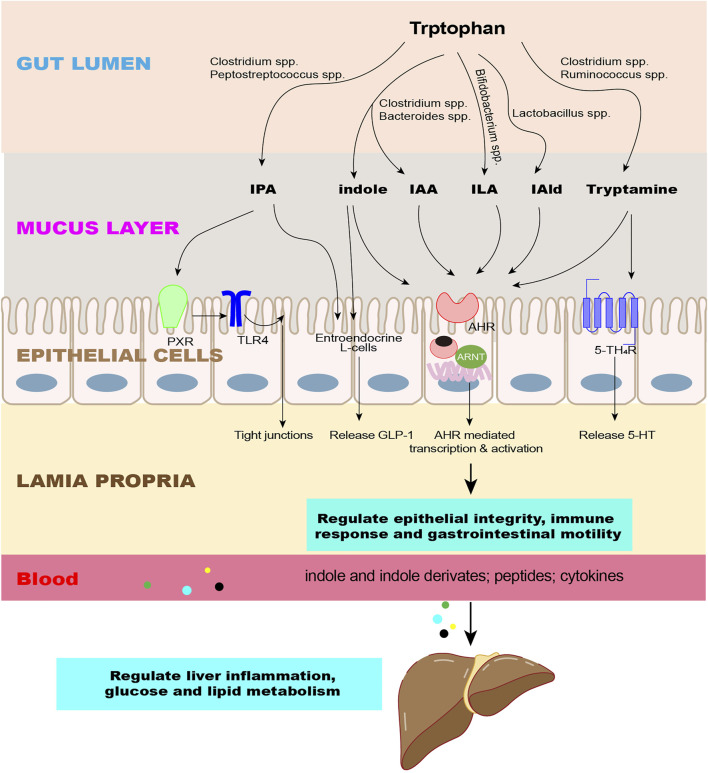
Effects of indole and its derivatives on the intestine and liver. The microbiota converts Trp into indole and its derivatives as signaling molecules to regulate epithelial integrity, immune response, and gastrointestinal motility through intestinal receptors and enter the liver through the circulation to regulate liver inflammation and glucose and lipid metabolism. Abbreviations: IPA, indole-3-propionate; IAA, indole-3-acetate; ILA, indole-3-lactate; IAld, indole-3-aldehyde; AhR, aromatic hydrocarbon receptor; PXR, pregnane X receptor; 5-HT_4_R, 5-HT4 receptors; GLP-1, glucagon-like peptide 1; TLR4, toll-like receptor 4.

The aromatic hydrocarbon receptor (AhR) is distributed in almost all tissues in various mammals and is expressed abundantly in the placenta, liver, and lungs, where it plays multiple roles in regulating the immune response, carcinogenesis, metabolic diseases, and neurophysiology (Xue et al., 2018; Girer et al., 2020; Vogel et al., 2020). In the gut, AhR is expressed in epithelial cells and immune cells (Nguyen et al., 2013; Ikuta et al., 2016). AhR is a ligand-inducible transcription factor that exists in the cytoplasm when it is at rest. After binding to the ligand, AhR translocates into the nucleus, where it heterodimerizes with the AhR nuclear transport protein (ARNT) and affects the expression of target genes, such as Cyp1a1, Cyp1b1, AhRR, and IL-10 (Lamas et al., 2018) (Figure 3)

A variety of indole-based compounds are ligands of AhR, differing in their pharmacological parameters. Using a reporter gene assay, a recent study found that microbial intestinal-catabolized indoles are full or partial agonists of human AhR. Indole, IAM, and IPyA are ligands with high efficacies of AhR; indole-3-ethanol (IEA), IA, skatole, and tryptamine have medium efficacy, but IPA, IAA, ILA, and IAld have no efficacy or low efficacy (Vyhlidalova et al., 2020b). Another study identified four new tryptophan metabolites that can activate AHR, which are 3-methyl-2-hydroxyindole, 5-hydroxyindole-3-acetic acid, 3-indole acrylic acid, and indole-3-carboxylic acid. The authors also found that human AHR is more effective at physiologically relevant concentrations of tryptophan metabolites than mice (Dong et al., 2020).

The immunomodulatory benefits of indole and its derivatives are partly based on the AhR-driven mechanisms in intestinal DCs, intraepithelial lymphocytes (IELs), and innate lymphoid cells (ILCs). As AhR ligands, indole and some derivatives act through the AhR/IL-22 axis (Zelante et al., 2013; Cervantes-Barragan et al., 2017). IL-22 plays a critical role in regulating epithelial integrity and the immune response, including intestinal stem cells (ISCs) and epithelial regeneration, intestinal barrier protection, antimicrobial defense, intestinal homeostasis, and shaping metabolism (Keir et al., 2020). By activating AHR in CD4^+^ T cells, ILA shapes CD4^+^CD8αα+ double-positive intraepithelial lymphocytes to have regulatory functions; other indole derivates have a similar effect (Cervantes-Barragan et al., 2017). The ILC3-derived IL-22 production was found to be reduced in an alcoholic liver disease mouse model; IAA supplementation restored the IL-22 level and protected the mice from ethanol-induced steatohepatitis (Hendrikx et al., 2019). IAld stimulates lamina propria lymphocytes (LPLs) to secrete IL-22 partially dependent on AhR and induces phosphorylation of STAT3 to accelerate intestinal epithelial proliferation, thereby restoring damaged intestinal mucosa (Hou et al., 2018). Indole-3-carbinol (I3C) is a natural plant product and a known ligand for AhR in many cruciferous vegetables. A recent study found that I3C reduces colitis by preventing microbial dysbiosis and increasing the abundance of butyrate-producing gram-positive bacteria in mice in an IL-22–dependent manner. Neutralization of IL-22 blocked the protective effect of I3C on colitis and prevents I3C from dysbiosis and butyrate-induced remission (Busbee et al., 2020).

Indole and its derivatives also promote intestinal immune homeostasis by activating AhR to protect the intestinal barrier. The activation of the AhR pathway in intestinal epithelial cells (IECs) is vital for protecting the stem cell niched and maintaining intestinal barrier integrity (Metidji et al., 2018). Recently, Scott et al. identified three bacterial metabolites of tryptophan, IEA, IPA, and IAAld, which protect against increased gut permeability and alleviated dextran sodium sulfate (DSS)-induced colitis in mice by maintaining the integrity of the intestinal barrier in an AhR-dependent manner (Scott et al., 2020). IA was reported to promote barrier function and immune tolerance in DSS-induced colitis mice, which is related to inducing the mRNA expression of the AhR target gene *Cyp1a1* in the intestinal epithelium and immune cells by IA (Wlodarska et al., 2017). Indole and its derivatives also promote the expression of IL-10 by activating AhR, and functional IL-10 signaling is associated with barrier function. IAld increases the proliferation of epithelial cells and promotes the differentiation of goblet cells, reversing the decline of intestinal barrier integrity and systemic inflammation caused by aging in geriatric mice. This effect increases the expression of the cytokine IL-10 *via* AhR but does not depend on the type I interferon or IL-22 signaling (Powell et al., 2020). In a T cell–mediated colitis mice model, oral administration of IPyA, a microbiota-derived AHR agonist, decreases the frequency of IFN-γ^+^ IL-10^-^ CD4^+^ T cells and increases that of IFN-γ^-^ IL-10^+^ CD4^+^ T cells in the colon lamina propria. IPyA attenuates the severity of colon inflammation in mice, but treatment with an AHR antagonist inhibited the anti-inflammatory effect of IPyA (Aoki et al., 2018).

### Activation of PXR

The pregnane X receptor (PXR) participates in drug, glucose, bile acid, and cholesterol metabolism, and is also essential in maintaining intestinal homeostasis and abrogating inflammation (Daujat-Chavanieu and Gerbal-Chaloin, 2020). There has been accumulating evidence of the beneficial effects of indole and its derivatives on intestinal barrier function based on the activation of PXR. Indole methylated at positions 1 and 2, which were reported ligands and partial agonists of human PXR, induced the expression of PXR-target genes, including *CYP3A4* and *MDR1*, *in vitro* (Vyhlidalova et al., 2020a). IPA is the most reported PXR receptor and is enteric microbiome-derived (Alexeev et al., 2018). As a ligand of PXR, IPA downregulates enterocyte inflammation cytokines and upregulates mRNA expression of junctional protein-coding genes *via* PXR and Toll-like Receptor 4 (Venkatesh et al., 2014). IPA protects the hematopoietic system and gastrointestinal tract injuries against acute radiation exposure *via* PXR/acyl-CoA–binding protein signaling (Xiao et al., 2020). There is an 88% overlap of the AhR and PXR activators; therefore, crosstalk exists between AhR and PXR signaling (Rasmussen et al., 2017). IPA was reported to exert its effects by activating AHR and PXR, and higher expression of AHR and PXR was inversely related to cancer cell proliferation and the stage and grade of the tumor (Sari et al., 2020). However, a recent report pointed out that IPA is not a direct ligand of PXR. Peter and his colleagues examined the effects of 10 known intestinal microbial metabolites and identified indole and IAM as PXR ligands and agonists. Indole and IAM induced the PXR-regulated genes CYP3A4 and MDR1 in human intestinal cancer cells and enhanced the binding of PXR to the MDR1 promoter, but IPA alone did not have the above biological effects. The authors analyzed the controversial results of IPA *in vivo* studies and found that IPA can greatly increase the intestinal anti-inflammatory effect *via* PXR in the presence of indole (Illes et al., 2020). This explanation is in line with the report that IPA in combination with indole significantly activated human PXR to regulate intestinal permeability and inflammation, but IPA alone had a weak agonistic effect (Venkatesh et al., 2014).

### Influence on Intestinal Hormone Release and Motility

Indole has a dual regulatory effect on the secretion of glucagon-like peptide 1 (GLP-1). Indole inhibits voltage-gated K+ channels and enhances Ca2+ entry to stimulate GLP-1 secretion acutely (6 min). However, over a longer period (240 min), indole slows ATP production, thus leading to a prolonged reduction in GLP-1 secretion (Chimerel et al., 2014). Tryptamine has been widely found to promote gastrointestinal motility. Tryptamine induces the release of 5-hydroxytryptamine (5-HT, serotonin) to modulate gastrointestinal motility as a neurotransmitter (Takaki et al., 1985; Mawe and Hoffman, 2013). Exogenous tryptamine can also act on 5-HT4R to increase anion and fluid secretion in the proximal colon and accelerate gastrointestinal transit (Bhattarai et al., 2018). Nevertheless, opposite effects reported that tryptamine significantly inhibits gastric emptying and acid secretion and reduces pepsin secretion (Bell and Webber, 1979).

### Influence on Bacterial Physiology

As a signaling molecule, indole influences diverse aspects of bacterial physiology, such as biofilm formation, spore formation, plasmid stability, bacterial motility, antibiotic resistance, and host cell invasion (Lee and Lee, 2010; Li and Young, 2013; Kim and Park, 2015). Intestinal bacteria have a dynamic and biphasic chemotaxis effect on indoles. It was reported that when the indole concentration is below 1 mM, *E. coli* is only observed to have a repellent-only response. When the indole concentration exceeds 1 mM, *E. coli* changes to an attraction response in a time-dependent manner. Therefore, indoles might be able to repel foreign invasion through differences in the adaptation status of bacteria and, at the same time, attract the growth of symbiotic bacteria that have adapted to indoles (Yang J. et al., 2020). The gut microbiota coordinates its behavior by sensing signals derived from the host or microbiota. Indole is sensed by the bacterial membrane-bound histidine sensor kinase (HK) CpxA. The enteric pathogens *E. coli* and *Citrobacter rodentium* in the lumen decrease their virulence and downregulate gene expression at the locus of the enterocyte effacement (LEE) pathogenicity island to adjust to a high concentration of indole, which in turn affects their attachment (Kumar and Sperandio, 2019). Although microbiota metabolites affect the composition and function of the intestinal microbiota and regulate host immunity, most of them still lack identification and have unknown functions. Another recent study focused on a family of indole-functionalized bacterial metabolites termed indolokines, and the authors found that such substances are widely present in different bacteria and mouse feces and elicit immune responses in both plants and humans, which indicates that relatively conservative defense strategies exist in the biology (Kim et al., 2020).

## Indole and Its Derivatives in Gastrointestinal and Liver Diseases

### Intestinal Inflammation

Indole and its derivatives are first produced in the gastrointestinal tract, and their perturbation has a great influence on gastrointestinal disorders. An analysis of serum samples from more than 500 IBD patients observed a negative correlation between Trp levels and disease activity (Nikolaus et al., 2017). Indole metabolism also alters in IBD patients. The fecal levels of Trp and IAA in patients with IBD are decreased, but the Kyn content is increased, which indicates that IBD patients have an obvious Trp–Kyn conversion but less intestinal Trp metabolism, which corresponds to healthy subjects inducing greater AHR activation than IBD patients in fecal samples (Lamas et al., 2016). Serum IPA was reported to decrease by nearly 60% in subjects with active UC compared with healthy controls, which can also serve as a biomarker of remission (Wlodarska et al., 2017; Alexeev et al., 2018). Necrotizing enterocolitis (NEC) is an intestinal necrotizing inflammatory disease that occurs in premature infants. An *in vitro* study found that ILA, secreted by *Bifidobacterium longum subsp infantis*, is anti-inflammatory by interacting with Toll-like receptor 4 (TLR4) and AHR to prevent the transcription of inflammatory cytokines (Meng et al., 2020).

### Alcoholic and Nonalcoholic Fatty Liver Diseases

Indole and several indole catabolic metabolite disorders have been confirmed by a large number of reports in metabolic liver diseases (Hendrikx and Schnabl, 2019). In a cohort of 137 NAFLD subjects, the circulating levels of indole were significantly lower than those of lean people and were negatively correlated with BMI (Ma et al., 2020). Compared with nonobese individuals, Trp metabolism translates more to Kyn but less to IAA in the feces of obese or diabetic patients (Laurans et al., 2018). In an HFD-induced obesity-associated NAFLD mouse model, the levels of IAA and tryptamine, two metabolites that have powerful anti-inflammatory responses, were decreased in both the liver and cecum (Krishnan et al., 2018). Reduced IPA levels in obese subjects have also been reported, which can be normalized by gastric bypass surgery (Jennis et al., 2018). Caseinolytic peptidase B protein homolog (ClpB) is a bacterial protein that is positively associated with IPA in human plasma and negatively associated with BMI, waist circumference, and total fat mass (Arnoriaga-Rodriguez et al., 2020). The expression of the *TnaA* gene in the gut microbiome was reduced in HFD-fed mice, which is accompanied by a reduction in plasma levels of indole and indoxyl sulfate. The reduction of indole and indoxyl sulfate repressed the expression of miR-181a and miR-181b in adipocytes of mice, contributing to the progression of obesity, IR, and WAT inflammation (Virtue et al., 2019).

## Ongoing Research of Indole and Its Derivatives as an Intervention

### Supplement With Related Strains

Indole and its derivatives may have therapeutic effects on gastrointestinal and liver disorders, and regulating dysbiosis to promote intestinal Trp metabolism homeostasis is both a goal and a means. *Streptococcus rosenbergii* has the *fldAIBC* gene cluster that produces IA. IA produced by commensal *Peptostreptococcus* species restores intestinal barrier function and suppresses inflammatory responses in DSS-induced colitis mice (Wlodarska et al., 2017). Inoculating *Clostridium sporogenes* into germ-free mice accompanied by L-Trp-supplement diets promoted the production of IPA to protect mice from DSS-induced colitis through PXR (Venkatesh et al., 2014). The role of *Lactobacillus reuteri D8* in protecting the intestinal mucosal barrier and activating intestinal epithelium proliferation depends on the production of IAld. Mice colonized with live *Lactobacillus reuteri D8* can ameliorate intestinal mucosa damage caused by DSS (Hou et al., 2018).

### Supplement With Indole and Its Derivates

Direct addition of indole and its derivatives is another common intervention. Mucin is an energy source for certain symbionts to promote their colonization and benefit the epithelial barrier. A recent study indicated that supplementation with IPA strengthens the mucus barrier against LPS-induced inflammation by increasing mucins *in vitro* (Beaumont et al., 2018; Li et al., 2021). In comparison with AhR ligand-deprived diets, dietary I3C supplementation drives the expression of the AhR repressor (AhRR) in intestinal immune cells of AhRR-reporter mice, strengthens intestinal barrier integrity, and lowers susceptibility to colitis (Schanz et al., 2020).

Indole and its derivatives are also involved in liver disease therapy. Oral administration of indole can suppress the NF- κ B pathway and reduce LPS-induced liver inflammation (Beaumont et al., 2018). Intestinal levels of IAA are reduced during ethanol-induced alcoholic fatty liver disease. Supplementation with IAA can rescue the reduced production of IL-22 by innate lymphoid cells, prevent translocation of bacteria to the liver, and reduce ethanol-induced steatohepatitis (Hendrikx et al., 2019). Oral gavage with IPA (20 mg/kg) can inhibit NF-kB signaling, correct gut dysbiosis and endotoxin leakage, and attenuate steatohepatitis and metabolic disorders in rats fed a high-fat diet (Zhao et al., 2019). The IPA-enriched diet also significantly lowered fasting blood glucose and plasma insulin levels as well as the HOMA index in rats, which served as a candidate for the treatment of metabolic disorders with insulin resistance (Abildgaard et al., 2018).

### Microbial Metabolite Mimicry and Synthetic Indole

Natural metabolites are potent and well-tolerated drugs; however, the short half-life, poor oral bioavailability, and ubiquitous action of indole derivates are the limitations for their suitability as drugs (Kumar et al., 2020). The microbial metabolic mimicry can expand the potential drug repertoire and overcome some of the defects of natural compounds (Xiao et al., 2020). Dvorak Z et al. synthesized the FKK series that mimics the docking of the natural indole and IPA with PXR. These newly designed compounds exhibited significant intestinal anti-inflammatory effects and were nontoxic compared with other known PXR xenobiotics (Dvorak et al., 2020). Screening indole and indazole compounds with diverse structures and combined computational and experimental studies, Chen et al. generated multiheterocyclic AHR agonists, PY109 and PY108, which displayed biostability and achieved a therapeutic effect at a very low dose (Chen et al., 2020). 6-Phosphofructo-2-kinase/fructose-2,6-biphosphatase 3 (PFKFB3) is a master regulatory gene of glycolysis. Indole supplementation reduces hepatic steatosis and inflammation in HFD-fed mice in a myeloid cell PFKFB3–dependent manner. Therefore, indole mimics or specific activation of PFKFB3 expression in macrophages could be feasible methods to prevent and treat inflammation-related diseases, such as NAFLD (Ma et al., 2020).

Recently, medicinal chemists have designed various active pharmacophores of indole analogs with antidiabetic and antidyslipidemic activities (Kumari and Singh, 2019). For example, a series of enantiomerically pure indole derivatives, 3a-rvia Friedel-Crafts alkylation of indole 1 with enones 2a-r, were designed, some of which were identified as potent inhibitors of α-glucosidase (IC50 = 4.3 ± 0.13–43.9 ± 0.51 μM); the activity is several folds higher than that of acarbose (IC50 = 840 ± 1.73 μM) (Islam et al., 2018). Compound 13 m is a hybrid derivative of indole and triazole, showing effective anti-lipogenesis activity with an IC-50 value of 1.67 μM. Compound 13 m improves dyslipidemia in HFD-fed hamsters by activating reverse cholesterol transport, which has a therapeutic tendency for the intervention of obesity and metabolic syndrome (Rajan et al., 2018). However, compared with the progress of the new synthetic indole molecules in the field of anticancer, antibacterial, or anti–Alzheimer’s disease, no antidiabetic and antihyperlipidemic active molecules of indole are currently under clinical trials.

### Genetic Manipulating

Apart from drug compounds, bacterial species engineering can be another effective avenue for modulating Trp metabolism. Genetic manipulation can transfer the gene of enzymes encoding Trp metabolism to some common and genetically tractable commensal. Codon optimizing and using a constitutively active phage-derived promoter to control the codon may be a good choice for facilitating robust expression in an exogenous host. IAA supplementation can restore the expression of IL-22 and have a protective effect on alcohol-induced steatohepatitis mice. Hendrikx et al. engineered *Lactobacillus reuteri* and found that supplementation of engineered probiotics can produce IL-22, reduce liver disease caused by chronic alcohol intake, and have clinical application prospects (Hendrikx et al., 2019). Compared with *Bacteroides thetaiotaomicron* Trp D-colonized mice, germ-free mice colonized with engineered *Bacteroides thetaiotaomicron* Trp D+ had significantly higher tryptamine levels to reduce DSS-induced epithelial barrier disruption and accelerate whole gut transit in mice (Bhattarai et al., 2018; Bhattarai et al., 2020). Lemon exosome-like nanoparticles (LELNs)-manipulated *Streptococcus thermophilus ST-21* and *Lactobacillus rhamnosus GG* probiotic mixture can decrease the mortality of *Clostridioides difficile* infection in mice partly by increasing the levels of ILA and indole-3-carboxaldehyde (I3Ald) to activate the AhR/IL-22 signaling. Meanwhile, the increased lactic acid inhibited the expression of indole biosynthesis gene *TnaA* to decrease the production of indole in an AhR-independent manner, both of which led to a decrease in *Clostridioides difficile* fecal shedding (Lei et al., 2020).

## Potential Side Effects of Indole and Its Derivates

### Intestinal Side Effects

According to reports on the potential side effects of indole and some derivatives under pathological conditions, some cautions have been raised. The pathogenic nature of bacteria may lead to infections, cancer, or even death. In weaned pigs, Trp supplementation negatively affects the small intestinal structure. Compared with Trp-free and low Trp diets, the piglets fed a high Trp diet significantly increased crypt depth and significantly decreased villus height to the crypt depth ratio (VH/CD) in the jejunum, and the mRNA expressions of the tight junction proteins, occludin and ZO-1, were also decreased. (Tossou et al., 2016). It has been reported that ILA supplementation impairs the effect of total paeoniflorin in the treatment of colitis in mice. Under the background of intestinal inflammation, the local accumulation of ILA could lead to the disruption of epithelial autophagy, increase the susceptibility of colon injury, and promote the progression of colitis in mice (Fan et al., 2020). Indole and its derivates can promote the expression of anti-inflammatory factor IL-22 to regulate intestinal homeostasis, but in the later stage of cancer, IL-22 may promote tumor progression (Hernandez et al., 2018). *Ruminococcus gnavus*, the tryptamine producer, is also an avid mucin degrader and only transplantation *R. gnavus* may lead to the progression of IBD (Henke et al., 2019). Several *Peptostreptococcus* species contain a gene cluster enabling the production of IA, but patients with bacteremia from *Peptostreptococcus* species have an increased risk of colorectal cancer (Kwong et al., 2018)*.* In addition, indole levels are increased in patients with *Clostridioides difficile* infection, and high indole levels might play a role in *Clostridioides difficile* proliferation by limiting the growth of beneficial indole-sensitive bacteria and altering the colonization resistance (Darkoh et al., 2019).

### Liver Side Effects

A Trp-enriched diet and indole intake could raise the risk of portal hypertension in patients with liver cirrhosis. As a common complication of liver cirrhosis, portal hypertension is associated with a poor prognosis. However, a lower dose (<10 mg/kg) of indole can influence portal blood pressure by increasing the portal blood flow. Both healthy rats and rats with portal hypertension induced by liver cirrhosis show an increased portal blood level after intracolonic administration of indole (Huc et al., 2018). A recent study found that in CCL4-induced liver fibrosis mice, oral IPA intervention (20 mg/kg/d) aggravated liver damage and fibrosis by activating HSCs *via* the TGF-β1/Smad signaling pathway (Liu et al., 2021). Indole and its derivates can activate PXR and/or AhR; however, there are reports that both activation and knockout of PXR exhibit an increase in lipid accumulation in hepatoma cell lines (Bitter et al., 2015). Similarly, overexpression of AhR in the liver leads to insulin resistance (Remillard and Bunce, 2002; Kumar et al., 2021). Low-affinity AhR allele-expressing mice are less sensitive to HFD-induced obesity and liver steatosis (Kerley-Hamilton et al., 2012). However, these reports did not directly use indole and its derivatives as interventions. Therefore, further research is needed to clarify the relationship between Trp metabolic derivatives and the function of liver AhR/PXR signaling under pathological conditions.

## Limitations and Perspectives

Increasing evidence has clarified the benefits of indole and its derivatives on immune homeostasis and metabolism. However, at present, the main reported bacterial genera are focused on *Clostridium, Lactobacillus, Bacteroides, Peptostreptococcus, Parabacteroides, Bifidobacterium, Escherichia,* and *Ruminococcus* (Gasaly et al., 2021), but bacterial metabolism is a much more complex and diverse process than we summarized above. Intestinal symbiotic bacteria express a variety of catalytic enzymes, and strains of the same bacterial species can also have different responses to environmental stressors (Zhao et al., 2018). The whole scenario of the related metabolism and the molecular mechanisms of physiology and pathology has not been fully revealed.

Currently, the emerging intervention tools offer a promising approach to therapeutic strategies, but the limitation of the technology is another obstacle. Take genetic engineering methods as an example. Enzymes derived from other species are heterologous and therefore may limit the high production of indole and its derivatives in human symbiotic bacteria. Compared to more common laboratory strains such as *E. coli*, there are relatively few genetic tools available for manipulating human symbiotic gut bacteria. In addition, in the context of the complex and dynamic gut ecosystem, heterologous enzymes may impair the adaptability of engineered bacteria. For example, the ratio of *Bacteroides*/Firmicutes in the intestines of NASH patients is significantly reduced (Sobhonslidsuk et al., 2018), so it may be difficult to ensure the therapeutic effect of engineered *Bacteroides thetaiotaomicron* transplanted to NAFLD patients. Therefore, it is necessary to promote the metabolism and stress tolerance of engineered bacteria.

The instability among existing research results also hinders further progress in drug development. The different doses also lead to different effects. *In vitro*, indole inhibits the release of GLP-1 at 0.3 mM, while enhancing GLP-1 release at 1 nM. It is not yet clear which concentration of indole effects is dominant *in vivo* (Chimerel et al., 2014). In addition, indole can activate AHR in most reports, but indole at 100–250 μM exhibits AHR antagonist activity, which inhibits TCDD-induced AhR activation and Cyp1a1 and Cyp1b1 expression in colonic crypts of mice (Jin et al., 2014). Therefore, a comprehensive understanding of the effects and causality of indole and its derivatives under pathological conditions is necessary to assist the development of individualized bacteriotherapy.
